# Oil of Turpentine in Incarcerated Herniæ

**Published:** 1829

**Authors:** 


32. *Oil of Turpentine in Incarcerated Herniæ.* Prof. Sewall, of the Federal city school, has published in the Am. Journal, the notes of a case of incarcerated scrotal hernia, which was cured by oil of turpentine taken internally. The following is his account of what took place under its administration:

I first attempted a reduction of the bowel by taxis, but as my exertions were unavailing, I bled him largely and then renewed my exertions, but without success. I then gave him two ounces of the spirit of turpentine, and instructed my pupils, who remained with him, to repeat the same dose every hour, till eight ounces were taken, or some sensible effect produced. Soon after I left him, a profuse sweat took place, and he fell into a tranquil sleep. In about two hours the herniæl tumour became soft and yielding and spotaneously retired from the scrotum. On repeating my visit in the middle of the day, I found he had taken about six ounces of the turpentine, and without experiencing any inconvenience from it. He was still sleeping, and entirely relieved. The next day he was at work in the brick-yard, and with no other complaint than that of a slight looseness of the bowels, and a scalding sensation in the rectum in passing his stools. No strangury was produced.—p. 301.

The professor acknowledges himself indebted for the practice to Dr. McWilliams, who had used the same medicine successfully in two cases.

